# Using a pedotransfer (PTF) model to establish GIS-based maps for the main physical and hydraulic soil properties in the eastern region of the Al-Ahsa Oasis, Saudi Arabia

**DOI:** 10.1371/journal.pone.0276259

**Published:** 2022-10-20

**Authors:** Abdullah Hassan Al-Saeedi

**Affiliations:** Department of Environmental and Natural Resources, College of Agricultural and Food Sciences, King Faisal University, Al-Hassa, Saudi Arabia; King Saud University, SAUDI ARABIA

## Abstract

This study aims to produce digital maps showing the physical and hydraulic soil properties of the Al-Ahsa Oasis in Saudi Arabia by employing the capabilities of the GIS technique. These maps can display the pattern distribution of different physical and hydraulic properties of soil accurately and accessibly. Recently developed local pedotransfer function (PTF) models were applied to the basic soil data of earlier research covering 566 points. An analysis was conducted using a spatial interpolation technique of the GIS program. Maps of spatial patterns described essential soil physical and hydraulic properties such as sand%, silt%, clay%, bulk density (*ρ*), saturation (θ_s_), field capacity (FC), wilting point (WP), and soil water characteristic curve (SWCC) fitting parameters *b*, *c*, *d*. Sand dominates most of the study area, particularly in the northeast near Hufof. This may be attributed to the deposition of drifting sand and dune movement. Silt and clay increased in other locations. Bulk density *ρ* was positively increased with sand and negatively with silt and CaCO_3_ content. Soil hydraulic properties (θ, FC, WP, and SWCC fitting parameters *b*, *c*, *d*) were positively correlated with silt and *ρ* and negatively with sand content. This digital map can be employed for a general overview investigation, for the whole studied area, for agricultural expansion and for environmental studies.

## 1. Introduction

Globally, there has been an increased demand for soil data and information for quantitative environmental monitoring and modeling [1]. To effectively manage food production, environment, and ecosystems at field, catchment, regional, and continental scales, it is crucial to have accurate, up-to-date, and spatially referenced information [2–4]. A geographic information system (GIS), by itself or associated with satellite images, is an effective tool for organizing, analyzing, and presenting the horizontal spatial variables of the soil properties and conditions [4–7]. Soil specialists, irrigation engineers, environmental engineers, and geotechnical engineers can use GIS maps for preliminary assessments and final geotechnical designs [4, 8–13]. GIS tools can be used for the following purposes: integrating existing data with project-specific data, identifying potential environmental and geological hazards, planning and tracking fieldwork, producing maps and figures, and improving communication [14, 15]. Early in the design process, these tools can assist in identifying potential barriers that may hinder project completion or agricultural production and avoid costly rework later on [8, 11, 12, 16, 17].

Hydraulic soil properties are integral to hydrological quantification, agriculture resource management, managing pollution transport, geotechnical engineering for construction and road building, and construction of water resource infrastructure, e.g., dam and irrigation and drainage projects [18–20]. Moreover, the soil-water characteristic curve (SWCC) is considered the first step in assessment and the single dominant factor governing changes in the behavior of saturated and unsaturated soils for these applications [21, 22].

The adoption of the GIS technique for drawing an overall map depicting all the hydraulic soil properties (i.e., saturation θ_s_, saturated hydraulic conductivity k_s_, field capacity FC, wilting point WP, and water holding capacity) and some other properties (compaction, soil penetration, etc.) have been reported in many studies during the last two decades [4, 12, 14–16, 23–29].

Al-Ahsa is one of the oldest agricultural settlements in the Arabian Peninsula, dating to 4000 B.C [30].

Today, Al-Ahsa is the largest agricultural area dominated by date palms, with more than three million trees [31]. Recent studies [32, 33] showed that the Al-Ahsa Oasis consists of two parts, the old and new oasis, with a total area of approximately 20,000 ha. It is L-shaped toward the north and east, of which 8200 ha are irrigated and divided into 25,000 farms. Therefore, this study employs the GIS technique to produce digital maps for the measured physical and hydraulic soil properties of the Al-Ahsa Oasis in Saudi Arabia. The resulting maps display the distribution and variability of soil physical and hydraulic properties accurately and easily accessible manner.

## 2. Material and methods

### 2.1. Study area

**Fig 1:**
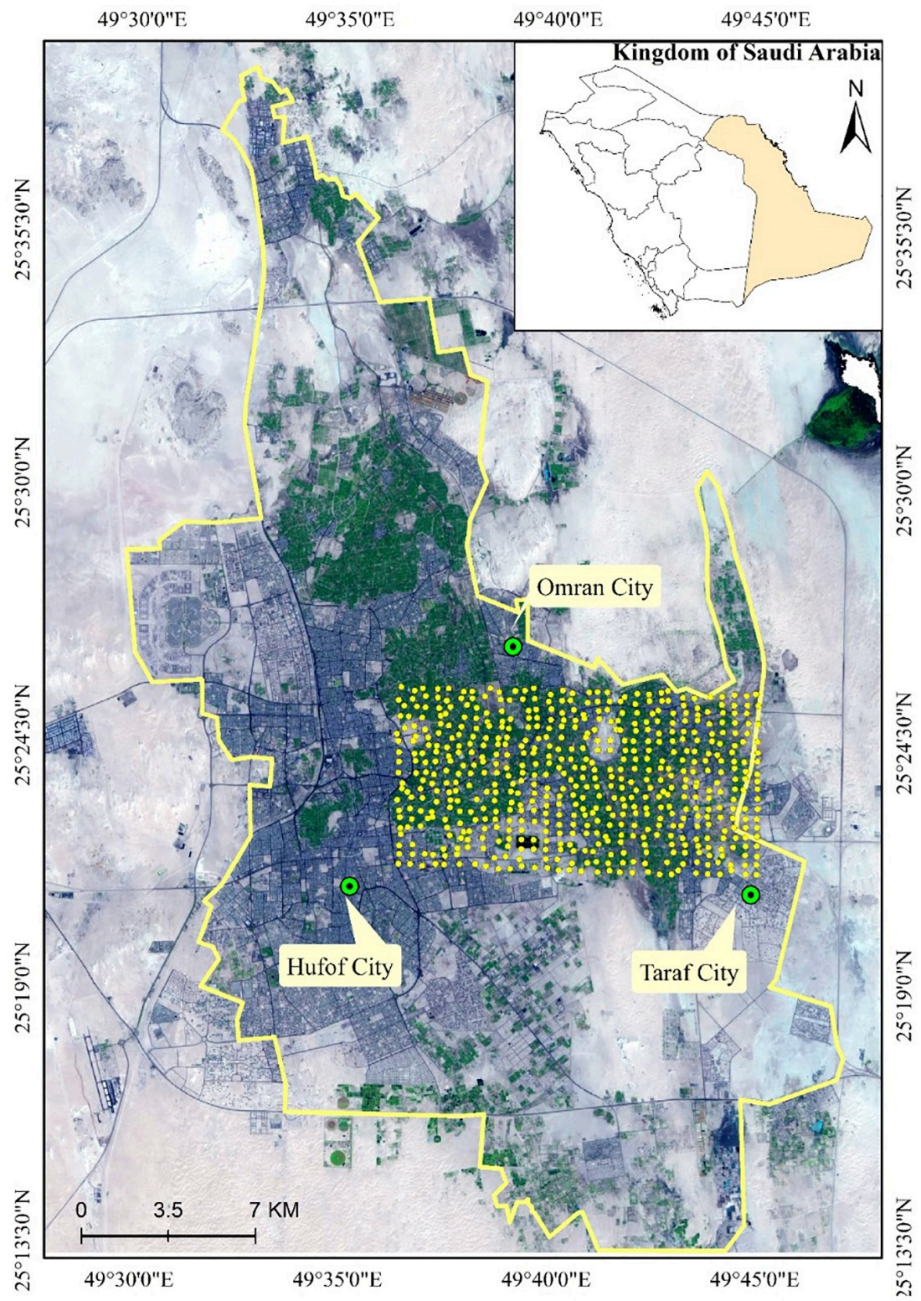
A general aerial image of Al-Ahsa shows its geographical position and sample locations U.S Geological Survey (USGS), 2022, Digital Orthophoto Quadrangles (DOQs): U.S. Geological Survey database, accessed November 17, 2021, at http://glovis.usgs.gov/app.) [39].

Al-Ahsa is located approximately 70 kilometers west of the Arabian Gulf, within latitude 25° 21′ and 25° 37′ N and longitude 49° 33′ and 49° 46′ E (Fig 1). The city of Hufof, the provincial capital, is situated in the southeast corner of the province at 150 meters above mean sea level (MAMSL). Towards the east and north, the slope drops to 105 MAMSL on the northern border and 122 MAMSL on the eastern border. Morphologically, the land surface of Al-Ahsa is flat except for the Al-Qarah Mountain, 13 km east of Hufof, which is composed of reddish-colored sedimentary rocks of the Upper Miocene to the Lower Pliocene Hufof Formation, which consists of calcareous sandstone, marl and clay.

The mountain has a flat top at 210 MAMSL, covering approximately 1400 hectares [34]. Outside the palm belt, the landscape is dominated by a mantle of eolian sand with a thickness of over 100 feet, interrupted by sabkhah and low mesa, the strata of which are nearly horizontal. The great northern sabkhahs, 6000 hectares, serve as catchment basins for highly saline drainage water that accumulates due to access irrigation [35]. The geological characteristics of Al-Ahsa outcrops consist of Tertiary and Quaternary period sedimentary rocks [34].

The pedogenesis and characteristics of soils in Al-Ahsa are determined by the extreme hot tropical climate, which evaporates the soil water and deposits the dissolved salts in the upper soil layers and across the whole profile as saline-sodic soil [36]. The soils of Al-Ahsa exhibit no profile development; they are composed of regosol deposits made up of transported particles. Six great groups of regosols can be identified, namely Salorthids, Gypsiorthids, Caliciorthids, Psammaquants, Haplargids, and Torripsaments [37]. The area’s climate is characterized by six hot, dry months in the summer and six months of cold and wet in winter. The air temperature can exceed 45°C during the summer, whereas it may reach 5°C in winter. An annual average of 50mm of rainfall falls primarily during the winter [38]. Al-Ahsa’s eastern part was the subject of this study, encompassing 12,860 hectares divided between a residential area and farmland.

### 2.2. Soil sampling and laboratory analysis

The original soil data (Table 1) were provided by Elprince et al. [40]. The study area was divided into 566 grid cells (380 m × 500 m) to establish a strategy for soil sample collection. Fifty randomly selected soil cores (0–20 cm depth) were crushed and mixed into one sample to collect a composite sample. Samples were air-dried, ground, thoroughly mixed, and passed through a 2 mm sieve to element gravels. Soil texture (percent sand, silt, and clay) was determined following the standard method of the hydrometer [41]. Soil bulk density (*ρ*) was determined using the well-tested local statistical equation (Eq 1) developed by Al-Saeedi (2022) [42] based on the percentage of silt.


$$\rho \left(g\;{cm}^{-3}\right)=1.5608-0.0064\times silt\%$$ (1)

**Table 1:** Summary statistics of the soil properties in the study area.

Statistic	*Clay*	*Silt*	*Sand*	*ρ*	θ_s_	FC	WP	*b*	*c*	*d*
	%	%	%	gm.cm^−3^	cm^3^.cm^−3^	cm^3^.cm^−3^	cm^3^.cm^−3^			
*n*	566	566	566	566	566	566	566	566	566	566
Minimum	1.000	2.000	45.500	1.574	0.171	0.076	0.055	8.876	2.210	0.056
Maximum	26.300	36.400	97.000	1.794	0.269	0.132	0.083	9.726	2.250	0.061
Mean	12.401	12.419	75.184	1.640	0.239	0.115	0.074	9.469	2.238	0.060
SD	3.899	7.788	10.108	0.050	0.022	0.013	0.006	0.193	0.009	0.001

*ρ*: bulk density, θ_s_: saturation, FC: field capacity, WP: wilting point, *b*, *c*, *d*: Eq 1 fitting parameters, n: number of observations, and SD: standard deviation.

### 2.3. Soil hydraulic properties and SWCC PTFs

This study addresses the major soil hydraulic properties. Soil water characteristics curve fitting parameters based on an earlier work developed a parametric logistic regression equation (PL4) (Eq 1) and local pedotransfer functions (PTFs) to predict soil moisture at any point of soil retention started from saturation θ_s_, field capacity FC, and wilting point WP (Eqs 2–8) [42]. The results provided excellent fitting and statistical significance [42]. The moisture content θ at any soil potential *ψ* is written as:

$$\theta (\psi )=c{\left(\frac{a-d}{\psi -d}\right)}^{\frac{1}{b}}$$ (2)

where *a*, *b*, *c*, and *d* are statistical fitting parameters; *a* is the maximum value on the *y*-axis or estimated moisture (θ) at zero suction (pF scale), which equals θ_s_.

Other soil parameters estimation equations:

$$\theta_{s}\left({cm}^{3}.{cm}^{-3}\right)=0.9668-0.4437\times \rho$$ (3)

$$FC\left({cm}^{3}.{cm}^{-3}\right)=-0.0210+0.5690\times \theta_{s}$$ (4)

$$WP\left({cm}^{3}.{cm}^{-3}\right)=0.0056+0.2877\times \theta_{s}$$ (5)

$$b=7.3872+8.7090\times \theta_{s}$$ (6)

$$c=2.3356-4.9210\times \theta_{s}+9.3802\times FC$$ (7)

d=0.0264-0.5938×FC+1.3663×WP (8)

### 2.4. GIS and interpolation method

GIS (geographic information system) is a computerized system with rules and procedures for analyzing and processing geographical information. These algorithms are utilized to link geographical data to their coordinates, group data into layers, and then display the data on a map showing the geographical characteristics of an area. Because each sample point contains its own spatial and geotechnical data, these data are arranged and tabulated in an Excel sheet in a manner compatible with the ArcMap 10.4 software program.

Interpolation is the estimation of a value within two known values in a sequence of values; in other words, it is a method for predicting the values of the grid at specific locations where there are no data samples [43]. Because of the high density of data in the study area, the inverse distance weighting (IDW) method is the most suitable for dense data interpolation [43]. IDW is an exact fast deterministic interpolator, estimating values at unmeasured points by a linear combination of values at nearby measured points [44]. The two-dimensional interpolation aims to determine the parameter *z* in non-scaled locations based on a specific set of parameter measurements at other sites *z*_i_. The parameter *z* represents any of the soil properties. The IDW algorithm is based on the equation:

$$Z_{i}=\frac{\sum_{i=1}^{N}Z_{i}.d_{i}^{-n}}{\sum_{i=1}^{N}d_{i}^{-n}}$$ (9)

where *Z*_*0*_ is the estimation value of the variable *z* in point *i*. *z*_*i*_ is the sample value in point *i*. *d*_*i*_ is the distance of the sample point to the estimated point. *N* is the coefficient that determines weight based on distance, and *n* is the total number of predictions for each validation case [45].

## 3. Results and discussion

### 3.1. Soil texture

**Fig 2:**
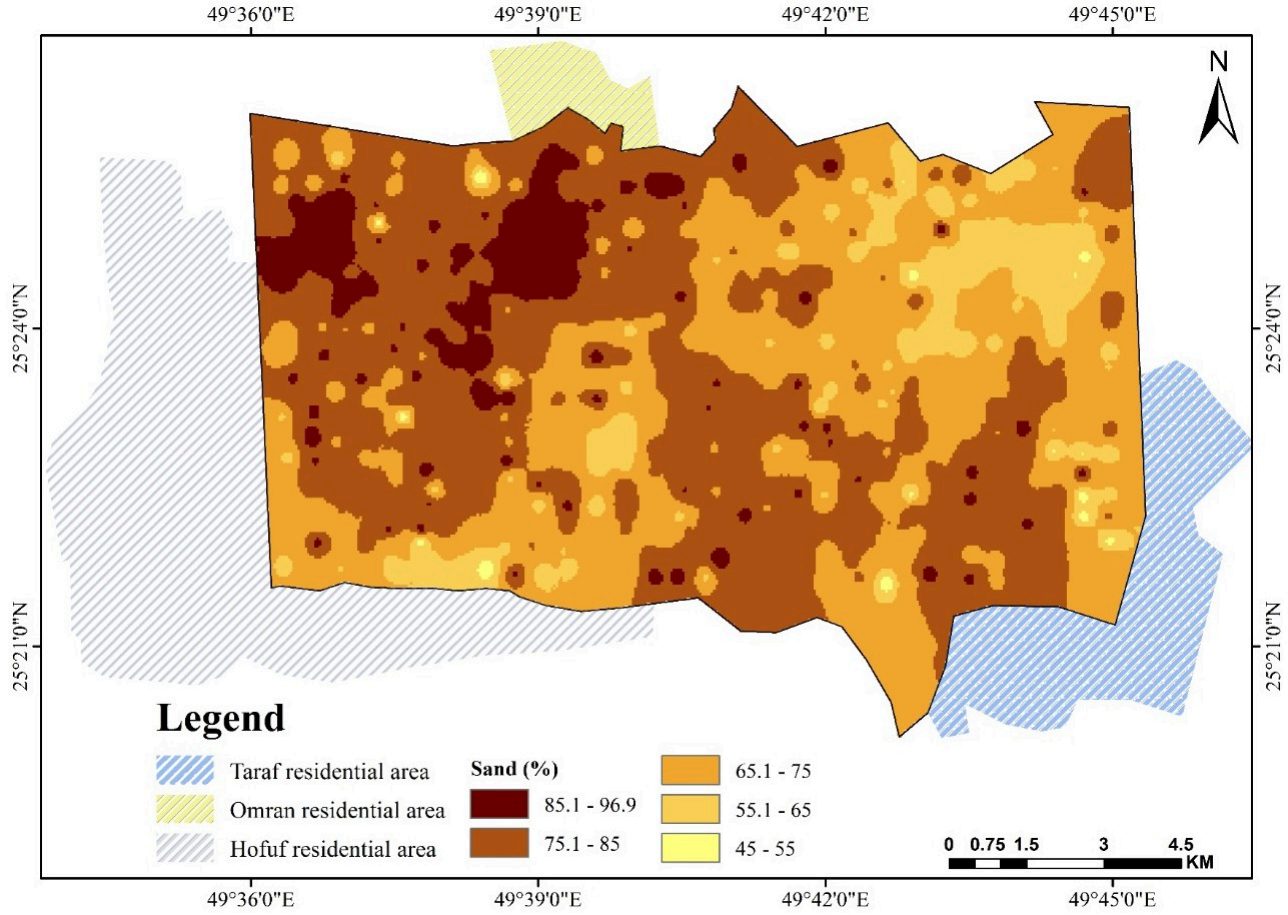
Spatial pattern of sand over Al-Ahsa.

Sandy soil covers only 10% of the study area, while sandy loam and loamy sand comprise more than 85% of the total area, with silty loam and loam making up about 5%. According to Fig 2, the sand percentage ranges from 45 to 96.9%. In the northeast corner of the study area, near Al-Omran city, toward Hufof, the highest concentration of sand particles exists. This may refer to the deposition of drifting sand and dune movement, horizontally creeping 10 to 15 m annually [46–48].

**Fig 3:**
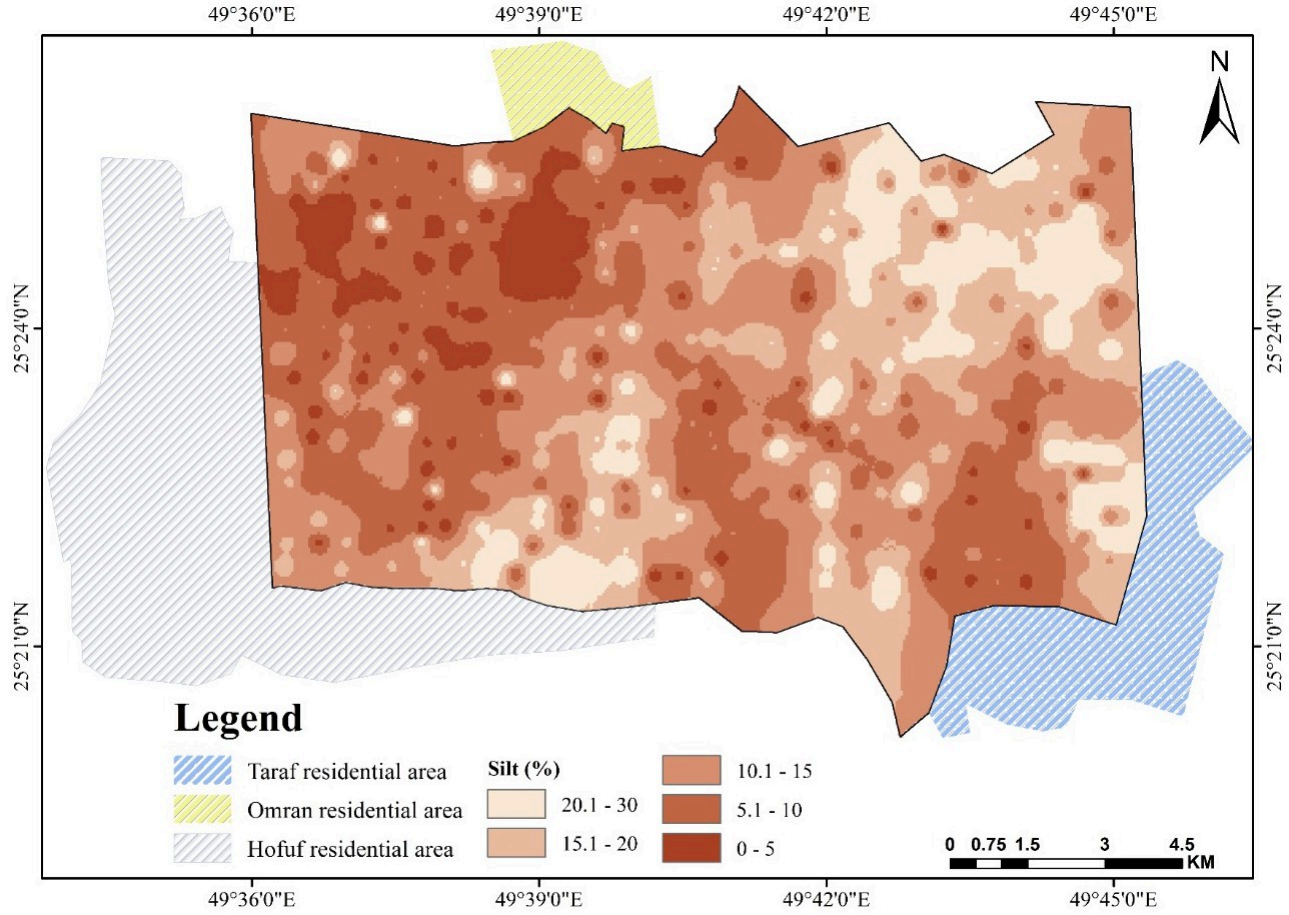
Spatial pattern of silt over Al-Ahsa.

**Fig 4:**
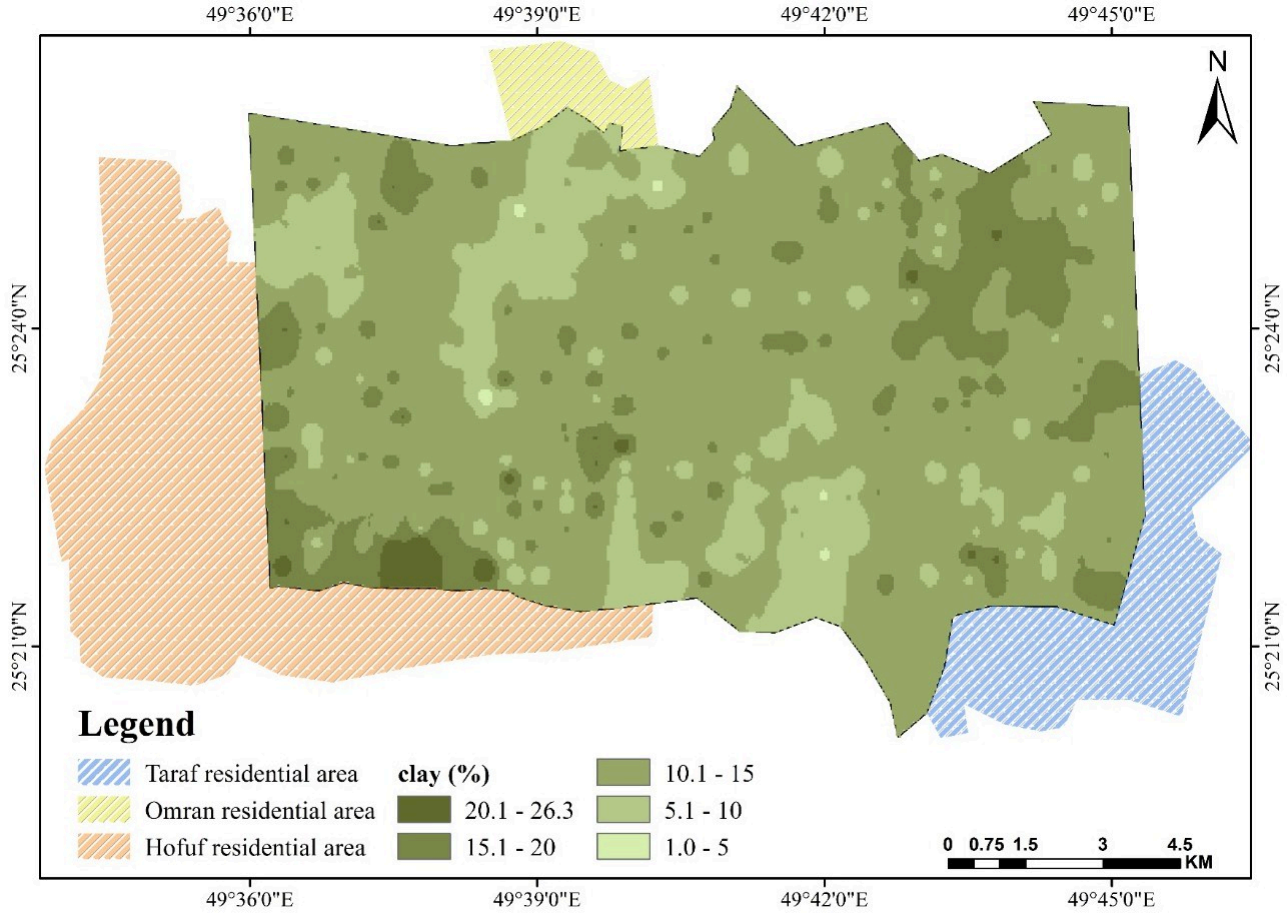
Spatial pattern of clay over Al-Ahsa.

The southwest part of the study area is also characterized by abundant sand deposits, which could be a continuation of the north wind effect and natural slope direction and aspect, as found by Elprince et al. [40]. The northwest corner and south showed the largest proportion of silt and clay where the area is not affected by sand drifting from the north. It is characterized by a relatively shallow soil profile with some influence of the shallow calcareous hardpan, which is rich in clay, silt, and CaCO_3_. Silt content shown in Fig 3 ranges from 30% to less than 5%, with the highest concentration in the northwest corner. As shown in Fig 4, the clay percentage varies from 20% in a very rare location to less than 5% [46, 49].

### 3.2. Bulk density *ρ*

**Fig 5:**
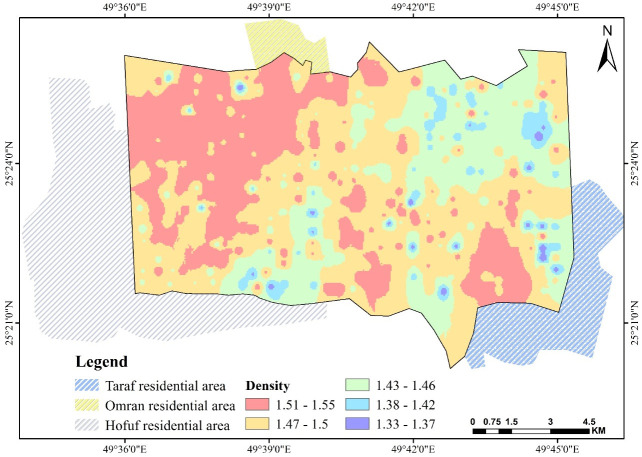
Spatial pattern of bulk density *ρ* over Al-Ahsa.

Fig 5, a density variation is correlated with the amount of sand and silt [42]. Areas with sandy texture soils, the northeastern corner and the southwest near Taraf, are characterized by densities ranging from 1.51 to 1.55 gm.cm^−3^. In the northwest corner, an area with a high silt content, the *ρ* value ranges between 1.46 and 1.43 gm.cm^−3^. In the locations where a high CaCO_3_ content is expected as high, especially near Al-Qara Mountain, due to the deposit of the solution of the mountain’s highly water-soluble rocks and south and southwest showed low *ρ* values, 1.33 to 1.37 gm.cm^−3^, which most probably due to the shallow sand layer and high CaCO_3_ percentage. The low density of CaCO_3_ can be referred to as the negative effects of CaCO_3_ content in soil on bulk density *ρ* values, which have been thoroughly studied and confirmed by numerous researchers [50, 51].

### 3.3. Saturation θ_s_

**Fig 6:**
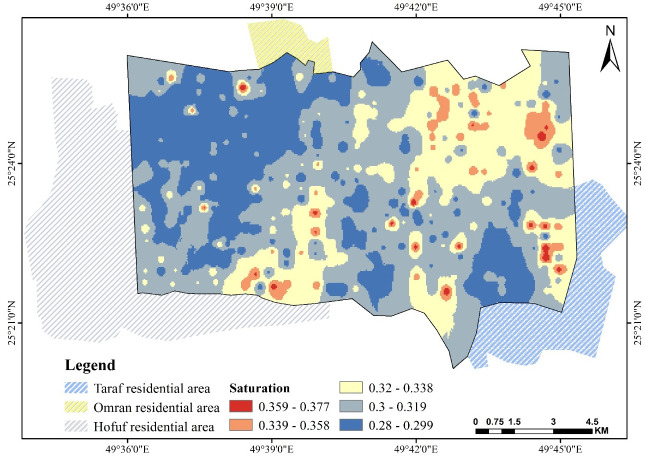
Spatial pattern of saturation θ_s_ over Al-Ahsa.

Saturation θ_s_ is highly affected by the ratio of pores and fine particles. Positive correlations always exhibited with silt% and clay% were due to increased pore ratio and, in contrast, a negative correlation for *ρ* and sand% [52–54]. As shown in Fig 6, θ_s_ ranges from 0.28 to 0.377 cm^3^.cm^−3^. A highly positive correlation of θ_s_ with the percentage of clay and silt is observed, which agrees with many other studies [52, 54, 55]. θ_s_ is generally low in oasis soils due to the dominant content of sand combined with rare clay [36, 42].

### 3.4. Field capacity FC and wilting point WP

**Fig 7:**
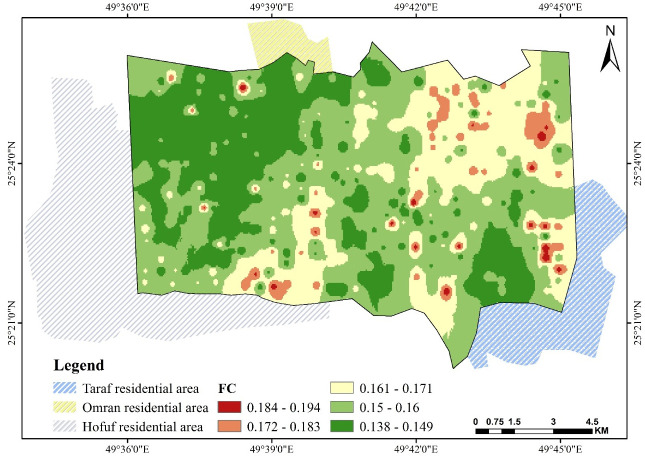
Spatial pattern of field capacity FC over Al-Ahsa.

**Fig 8:**
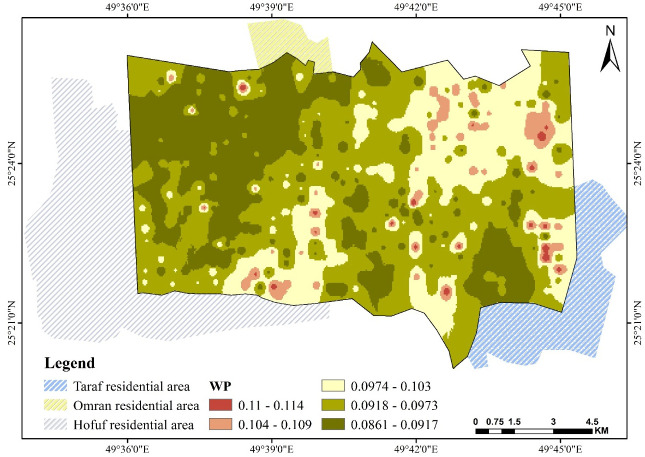
Spatial pattern of wilting point WP over Al-Ahsa.

Figs 7 and 8 show water content distribution at FC and WP. FC ranges between 0.194 and 0.138 cm^3^.cm^−3^, where the higher values are found at scattered locations in the studied area, while low contents are found in the area extending within the northwest corner. In these areas, the silt fraction is dominant over sand. The same observation is made with WP. Both properties are negatively related to the value of sand and positively related to silt and saturation θ_s_—the pore volume ratio increases as the fine particles, silt, and clay increase. The soil body will store more moisture at FC and WP. Several publications have reported this relationship, showing a negative correlation with sand and a positive one with silt and clay [56, 57].

### 3.5. Parameters *b*, *c*, and *d*

**Fig 9:**
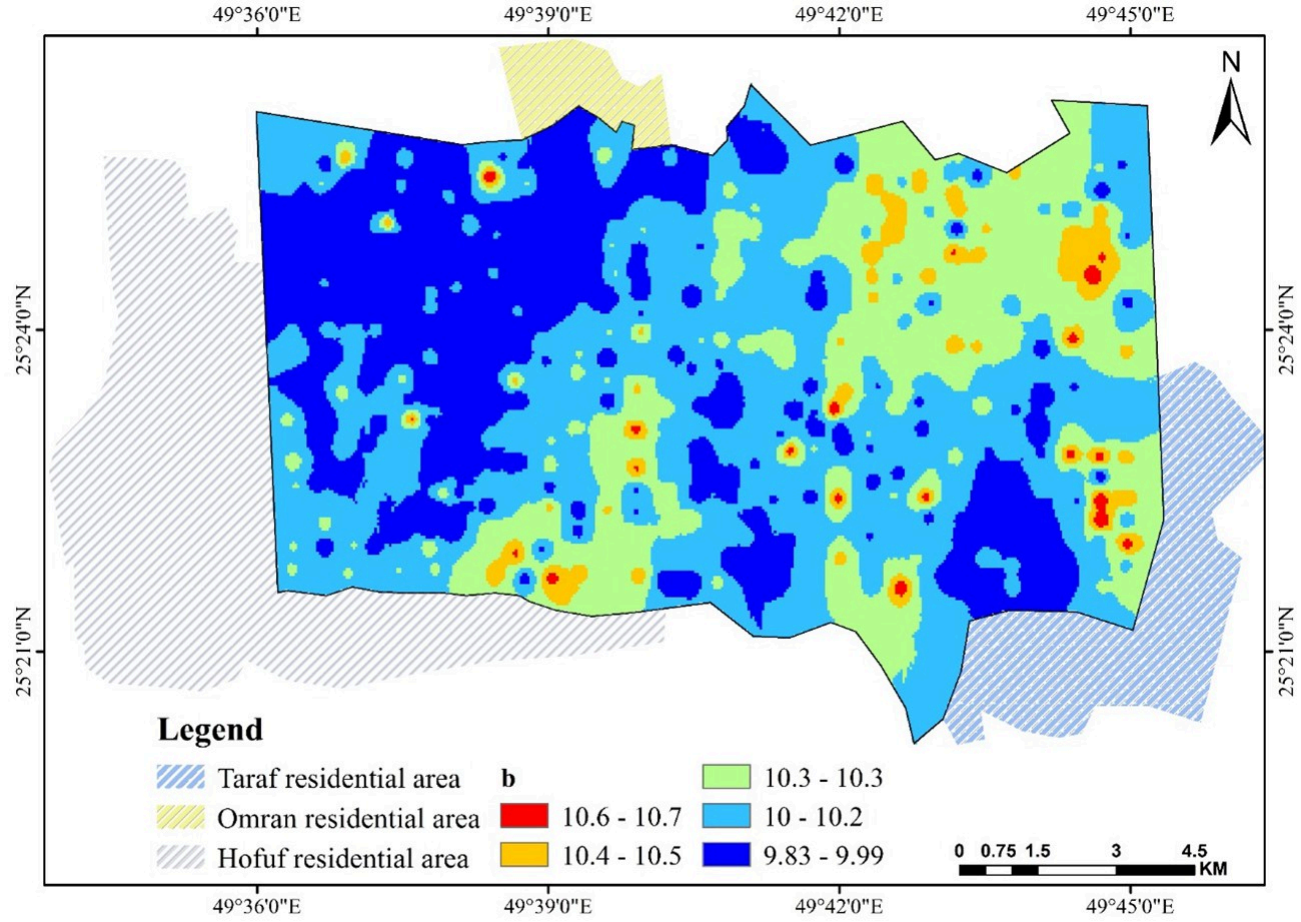
Spatial pattern of *b* over Al-Ahsa.

**Fig 10:**
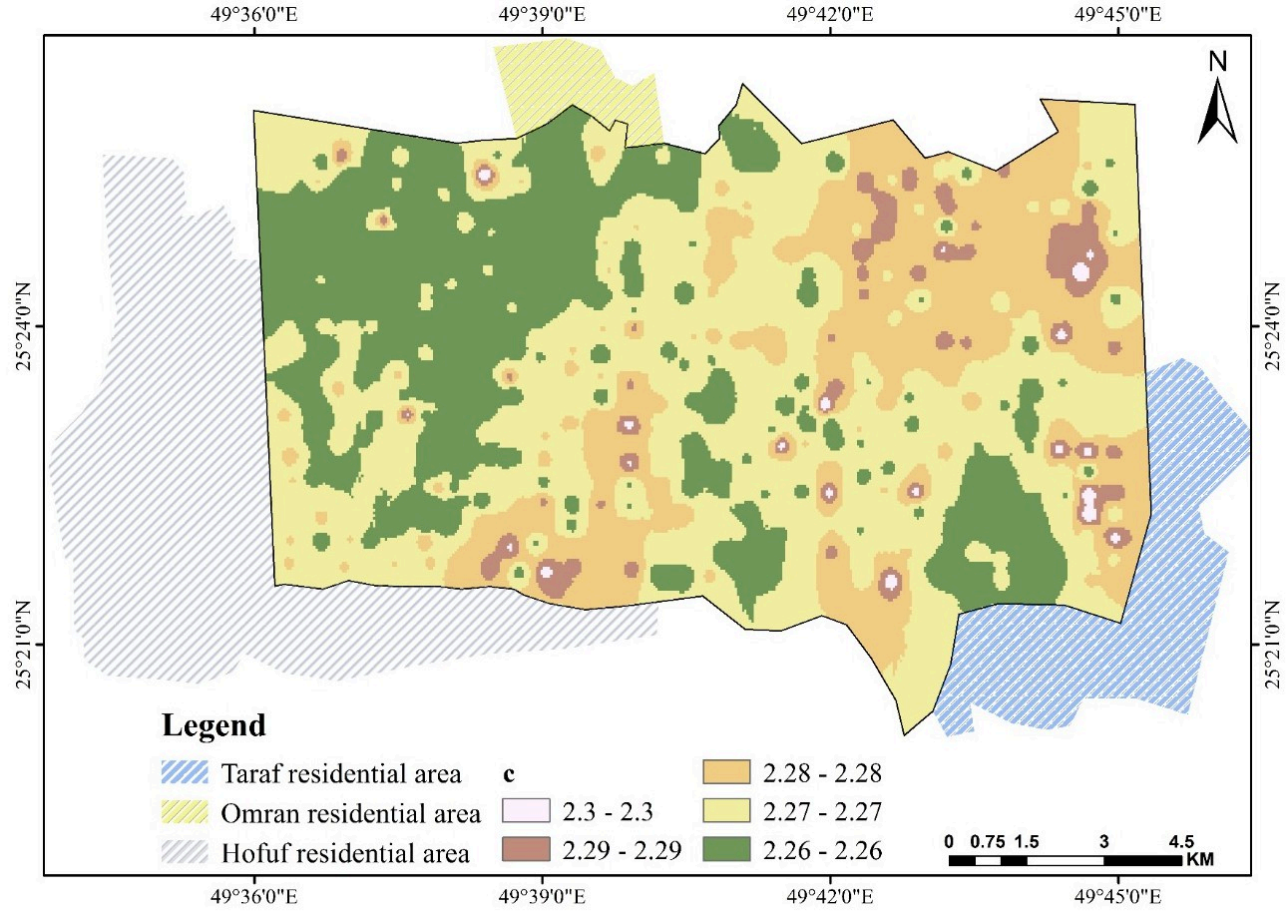
Spatial pattern of *c* over Al-Ahsa.

**Fig 11:**
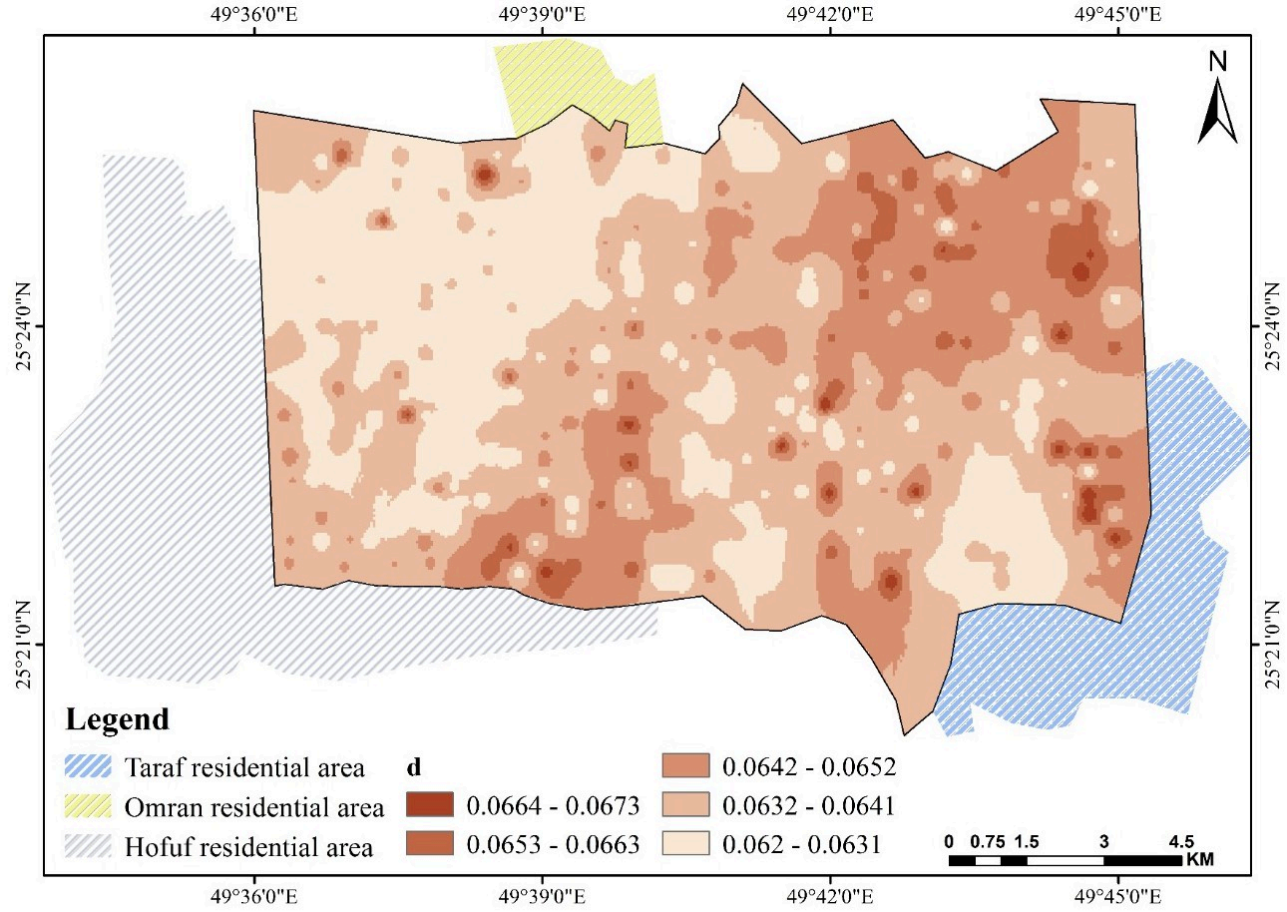
Spatial pattern of *d* over Al-Ahsa.

Eq (1) is based on the value of (*a*), equal to θ_s_; thus, all factors affect saturation θ_s_, consequently affecting these fitting parameters. Figs 9–11 show the fitting parameter values in Eq (1). These results support the findings of another study [55]. A good illustration of SWCC can be made by applying Eq (1) to the map information at any given location, using the simple but robust GIS algebra function, which can be used to perform any spatial correspondence, relation, and geographic analysis.

## 4. Conclusions

GIS-based digital maps can provide field researchers with a rapid method for determining the SWCC and other physical and hydraulic soil properties at any location while maintaining high accuracy. The soil texture of the study area is dominated by sandy loam and loamy sand with θ_s_ less than 0.300 cm^3^.cm^−3^, FC less than 0.160 cm^3^.cm^−3^, and WP less than 0.098 cm^3^.cm^−3^. Eq (1) parameter maps will be an excellent tool for estimating SWCC for agricultural and geotechnical purposes. A detailed study is necessary to verify the accuracy of the overall values depicted on the maps.

## Supporting information

S1 Data (XLSX)
